# Examining Prevalence and Diversity of Tick-Borne Pathogens in Questing Ixodes pacificus Ticks in California

**DOI:** 10.1128/AEM.00319-21

**Published:** 2021-06-11

**Authors:** Daniel J. Salkeld, Danielle M. Lagana, Julie Wachara, W. Tanner Porter, Nathan C. Nieto

**Affiliations:** a Department of Biology, Colorado State University, Fort Collins, Colorado, USA; b Colorado School of Public Health, Colorado State University, Fort Collins, Colorado, USA; c Department of Biological Sciences, Northern Arizona University, Flagstaff, Arizona, USA; Norwegian University of Life Sciences

**Keywords:** *Borrelia miyamotoi*, *Borrelia burgdorferi*, *Anaplasma phagocytophilum*, tick-borne disease surveillance, aggregated data

## Abstract

Tick-borne diseases in California include Lyme disease (caused by Borrelia burgdorferi), infections with Borrelia miyamotoi, and human granulocytic anaplasmosis (caused by Anaplasma phagocytophilum). We surveyed multiple sites and habitats (woodland, grassland, and coastal chaparral) in California to describe spatial patterns of tick-borne pathogen prevalence in western black-legged ticks (Ixodes pacificus). We found that several species of *Borrelia—*B. burgdorferi, Borrelia americana, and Borrelia bissettiae—were observed in habitats, such as coastal chaparral, that do not harbor obvious reservoir host candidates. Describing tick-borne pathogen prevalence is strongly influenced by the scale of surveillance: aggregating data from individual sites to match jurisdictional boundaries (e.g., county or state) can lower the reported infection prevalence. Considering multiple pathogen species in the same habitat allows a more cohesive interpretation of local pathogen occurrence.

**IMPORTANCE** Understanding the local host ecology and prevalence of zoonotic diseases is vital for public health. Using tick-borne diseases in California, we show that there is often a bias to our understanding and that studies tend to focus on particular habitats, e.g., Lyme disease in oak woodlands. Other habitats may harbor a surprising diversity of tick-borne pathogens but have been neglected, e.g., coastal chaparral. Explaining pathogen prevalence requires descriptions of data on a local scale; otherwise, aggregating the data can misrepresent the local dynamics of tick-borne diseases.

## INTRODUCTION

In California, the archetypal habitat-host system for natural Lyme disease transmission dynamics is the oak woodland of the northwest—particularly in Mendocino County—where western gray squirrels (Sciurus griseus) are the predominant reservoir hosts for the disease agent Borrelia burgdorferi *sensu stricto* (1–6). The western fence lizard (Sceloporus occidentalis) is also an important host of the western black-legged tick (Ixodes pacificus) vector, though borreliacidal blood factors mean that the lizard host removes B. burgdorferi from ticks and does not contribute to further B. burgdorferi transmission (5, 7–9). Lyme disease incidence is high in areas of northwestern California and can surpass 50 cases per 100,000 person-years (10).

However, research beyond Mendocino County oak woodlands has illuminated multiple other tick-pathogen disease systems across California’s diverse habitats. For example, I. pacificus has also been found infected with Borrelia miyamotoi*—*a spirochete that has been strongly implicated as a cause of human disease in California (11). State-wide surveillance for this pathogen shows that it is present in many of the same counties as B. burgdorferi (12). In the northeastern United States, B. miyamotoi prevalence in ticks is normally lower than that of B. burgdorferi from the same locations (13), but in California the relationship is less predictable. State-level observations, and observations in some counties (e.g., Alameda County), suggest that prevalence of the two *Borrelia* species is roughly equivalent in adult I. pacificus ticks but that B. burgdorferi *sensu lato* is more common in nymphal ticks (12, 14). However, patterns of the relative frequencies of B. miyamotoi-B. burgdorferi infection appear idiosyncratic, and sometimes B. miyamotoi can be the more frequent or the only spirochete in questing tick populations (12, 15–17). Importantly, B. miyamotoi can be vertically transmitted from mother to offspring, so questing larvae may also be infected (18, 19).

Other documented species of the B. burgdorferi *sensu lato* complex in California include Borrelia bissettiae and Borrelia americana. *B. bissettiae* has been observed in human sera in Mendocino County, though its pathological impact is uncertain (20). In California, *B. bissettiae* has been reported from a diverse array of mammals, including wood rats (*Neotoma* spp.), mice (*Peromyscus* spp. and Reithrodontomys megalotis), chipmunks (*Neotamias* spp.), and rats (Rattus rattus), and in both I. pacificus and Ixodes spinipalpis (3, 21–27) (Table 1). *B. americana* has been observed in I. pacificus and *I. spinipalpis*; human infections have not been reported (26, 27). Anaplasma phagocytophilum, which causes human granulocytic anaplasmosis, also occurs in western black-legged tick populations of northern California and has been observed in a variety of habitats (28, 29).

**TABLE 1:** Summary of geographic observations of Borrelia americana and Borrelia bissettiae in California

*Borrelia* species	County (site)	Tick species	Mammal species	Reference or source
Borrelia americana	Los Angeles (Malibu Creek)	I. pacificus		30
	Marin (Tennessee Valley and Owl Trail)	I. pacificus		This study
	Orange (Crystal Cove State Beach)	*I. spinipalpis*		26
	San Mateo (Windy Hill OSP)	I. pacificus		26
	Santa Barbara (Coal Oil Point Reserve)	*I. spinipalpis*		27

Borrelia bissettiae	Alameda	I. pacificus	Neotoma fuscipes (dusky-footed woodrat), Rattus rattus (black rat)	14, 23
	Contra Costa	Ixodes auritulus		15
	Del Norte	I. pacificus		23
	Humboldt		Neotamias senex (Allen's chipmunk), *Neotoma fuscipes*	24
	Marin (Fort Baker)	I. pacificus		26
	Mendocino	*I. spinipalpis* ex *N. fuscipes*, I. pacificus	Microtus californicus (California vole), *Neotoma fuscipes,* Peromyscus boylii (brush mouse), P. maniculatus (deer mouse), P. truei (pinyon mouse)	3, 23
	Monterey (Andrew Molera SP)	I. pacificus		This study
	Orange (Crystal Cove State Beach)	*I. spinipalpis*		26
	San Luis Obispo		Neotoma lepida (desert woodrat), *P. boylii*	21
	San Mateo (Thornewood OSP)	I. pacificus	*P. boylii*	25, 26
	Santa Barbara (Coal Oil Point Reserve, Paradise Reserve)	Ixodes peromysci, *I. spinipalpis*	*R. rattus, Reithrodontomys megalotis* (western harvest mouse), *P. maniculatus*	27
	Santa Clara (Foothills Park)		*P. truei*	25
	Santa Cruz (Wilder Ranch SP)	I. pacificus		26

**FIG 1:**
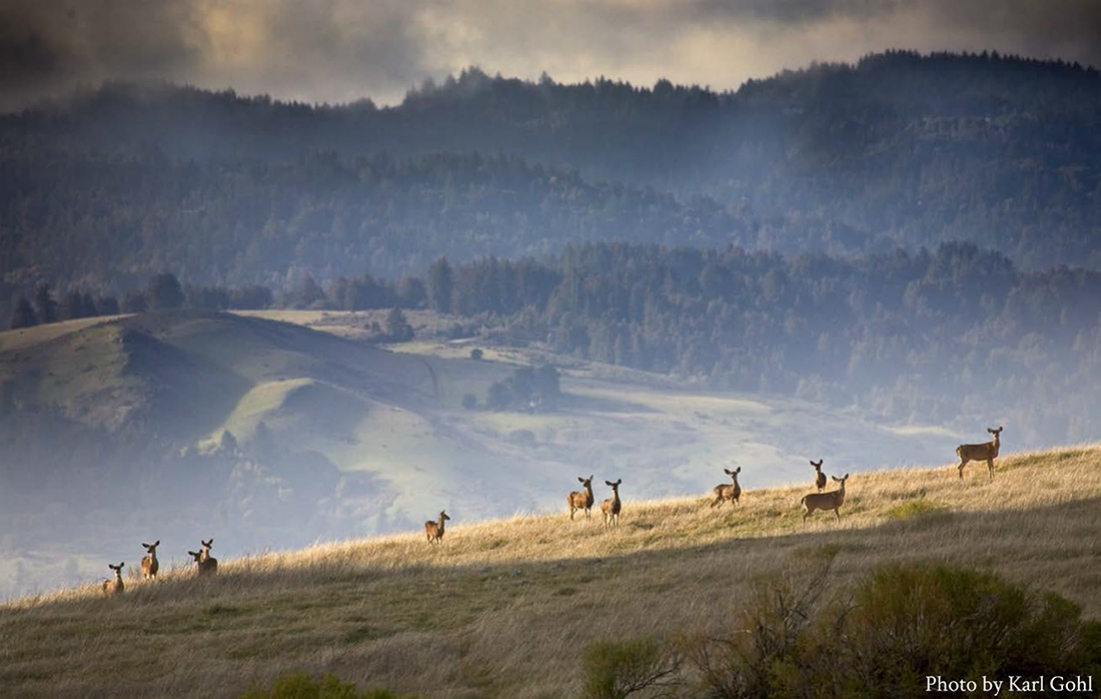
Deer in Monte Bello Open Space Preserve, illustrating the habitat heterogeneity of California’s landscape: a mosaic of grassland, chaparral, and woodland. (Courtesy of Karl Gohl; reprinted with permission.)

Understanding the host ecology (identifying the species that act as reservoirs and the habitat associations) and the human epidemiology (where, when, and how often people are exposed and whether the bacteria cause illness) of these different tick-borne pathogens is not simple. The landscape is diverse, including chaparral, oak woodland, grasslands, and redwood forest within a county’s limits and sometimes on the same hiking trail, with the implication that the reservoir host communities are also heterogeneous (Fig. 1). Consequently, the risk of exposure to tick-borne pathogens is geographically varied (14, 17, 26), and it is not always straightforward to describe local tick-borne pathogen prevalence. Should the infection prevalence in tick populations be described for a single trail, at the county level, or at a regional or state level? And what information is lost if the data are aggregated across these different scales?

Here, we examined the prevalence patterns of B. burgdorferi, *B. americana*, B. miyamotoi, *B. bissettiae*, and A. phagocytophilum in questing I. pacificus ticks at sites in coastal counties of central and northern California. We also explored the impacts of aggregating data from site to regional levels.

## RESULTS

### B. burgdorferi *sensu lato* prevalence in adult ticks.

**FIG 2:**
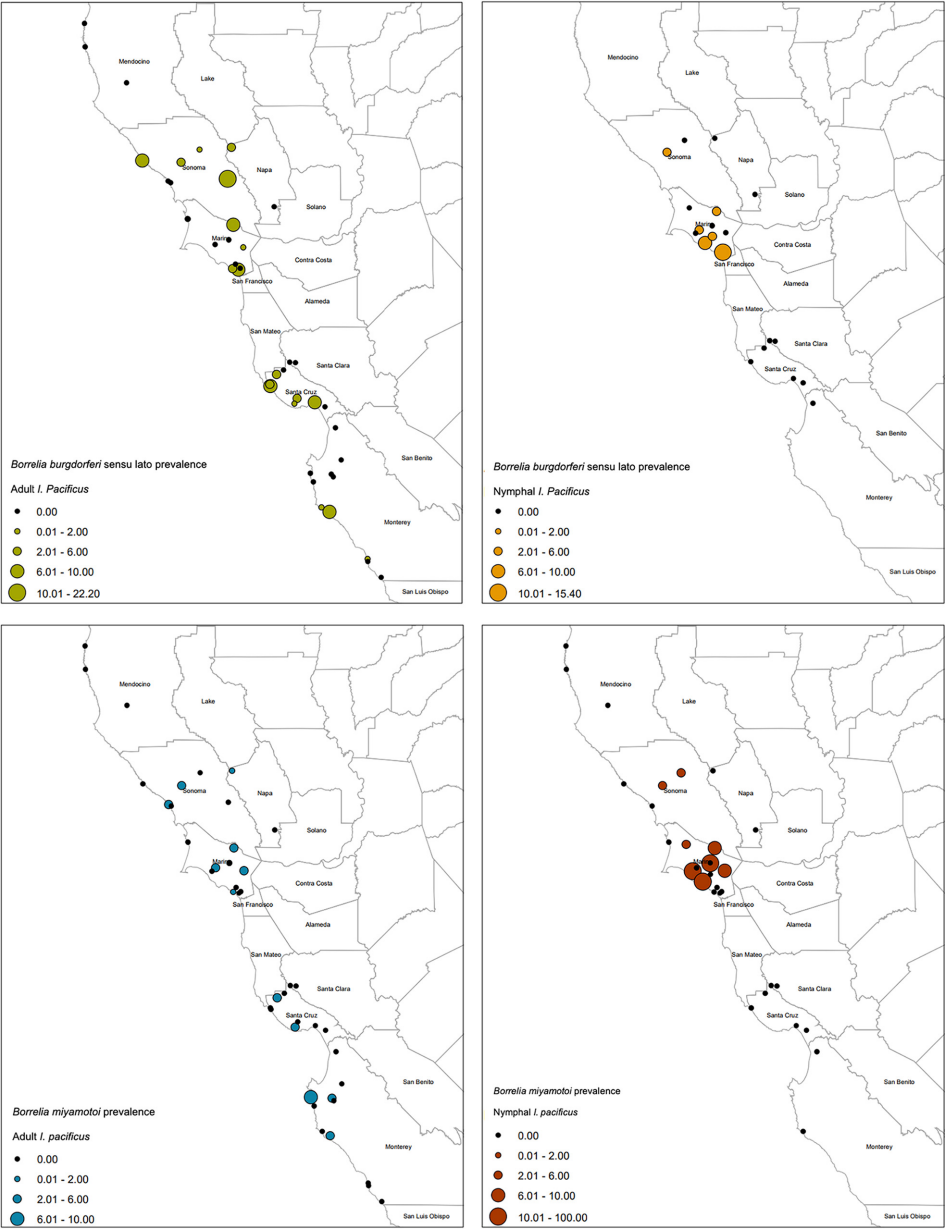
Maps showing collection sites for western black-legged ticks (Ixodes pacificus) and the infection prevalence (percentage positive) of Borrelia burgdorferi *sensu lato* (i.e., including B. burgdorferi *sensu stricto*, *B. americana*, and *B. bissettiae*) and Borrelia miyamotoi. The maps were created in ArcMap, and the polygon feature class of the California county boundaries was downloaded from ArcGIS (credits: U.S. Bureau of Reclamation, California Department of Conservation, California Department of Fish and Game, California Department of Forestry and Fire Protection, and National Oceanic and Atmospheric Administration).

**FIG 3:**
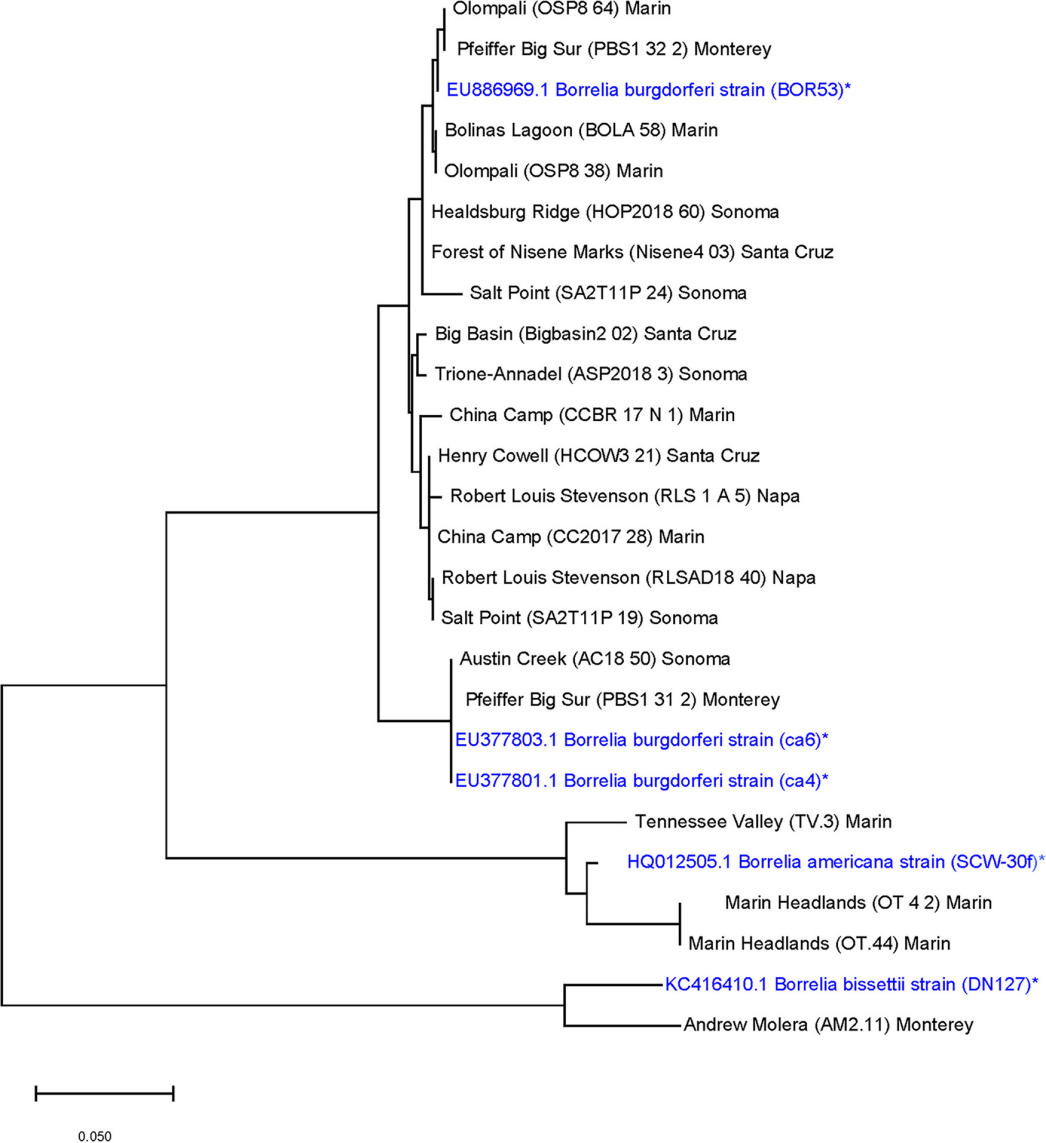
Maximum-likelihood tree and Tamura-Nei model demonstrating the relationship between previously and newly characterized sequences of the *Borrelia* 16S-23S intergenic spacer (IGS; *rrs-rrlA*). Newly characterized sequences were isolated from Ixodes pacificus. Blue sequences are previously characterized sequences. The tree is drawn to scale, with branch lengths measured in number of substitutions per site.

Collection sites and infection prevalence for B. burgdorferi *sensu lato* and Borrelia miyamotoi are shown in Fig. 2. Aggregated across all sites, real-time PCR prevalence of B. burgdorferi *sensu lato* was 2.9% (95% confidence interval [CI] = 2.3 to 3.7%) in adult ticks (Table 2). A total of 36 B. burgdorferi *sensu lato* samples were successfully sequenced from adult ticks, of which 32 (89%) were B. burgdorferi *sensu stricto* (Table 3). Sequencing also identified the presence of *B. americana* (*n* = 3) and *B. bissettiae* (*n* = 1) (Table 3; Fig. 3); further description of these results is below. Adult tick populations from Marin, Monterey, Napa, Sonoma, and Santa Cruz counties all harbored B. burgdorferi *sensu stricto* (sample sizes were >73 for each county). We did not observe B. burgdorferi *sensu lato* in adult ticks collected in Mendocino or Santa Clara counties, though samples from these two counties were small (*n* < 24 for both counties), and ticks were predominantly collected from coastal grassland or chaparral habitats in Mendocino County.

**TABLE 2:** County, regional, and state-wide reports of prevalence of Borrelia burgdorferi *sensu lato* and B. miyamotoi in questing adult and nymphal Ixodes pacificus ticks in California

Level of data aggregation	Prevalence^*a*^				Reference
	Adult ticks		Nymphal ticks		
	B. burgdorferi *sensu lato*	B. miyamotoi	B. burgdorferi *sensu lato*	B. miyamotoi	
Alameda County	29/3,070 (0.9, 0.6–1.4)	13/3,070 (0.4, 0.2–0.7)	189/2,890 (6.5, 5.7–7.5)	11/2,890 (0.4, 0.2–0.7)	14
	3/285 (1.1, 0.2–3.0)	0/73 (0, 0–4.9)	3/342 (0.9, 0.2–2.5)		12, 26
Marin County	42/1,039 (4.0, 2.9–5.4)	17/1,039 (1.6, 1.0–2.6)	17/483 (3.5, 2.1–5.6)	33/483 (6.8, 4.7–9.5)	This study
	14/682 (2.1, 1.1–3.4)	1/406 (0.2, 0.01–1.4)	24/331 (7.3, 4.7–10.6)	2/240 (0.8, 0.1–3.0)	12, 26
Mendocino County	0/23 (0, 0–14.8)	0/23 (0, 0–14.8)			This study
	0/61 (0, 0–5.9)	0/54 (0, 0–6.6)	0/19 (0, 0–17.6)	0/17 (0, 0–19.5)	12, 26
Monterey County	5/693 (0.7, 0.2–1.7)	5/693 (0.7, 0.2–1.7)	0/1 (0, 0–97.5)	0/1 (0, 0–97.5)	This study
	0/140 (0, 0–2.6)	0/49 (0, 0–7.3)	0/35 (0, 0–10.0)	0/2 (0, 0–84.2)	12, 26
Napa County	4/73 (5.5, 1.5–13.4	1/73 (1.4, 0.03–7.4)	0/20 (0, 0–16.8)	0/20 (0, 0–16.8)	This study
	3/285 (1.1, 0.2–3.0)	0/1 (0, 0–97.5)	3/342 (0.9, 0.2–2.5)	1/101 (1.0, 0.03–5.4)	12, 26
San Mateo County	1/86 (1.2, 0.03–6.3)	1/86 (1.2, 0.03–6.3)	5/203 (2.5, 0.8–5.7)	7/203 (3.4, 1.4–7.0)	17^*b*^
	15/620 (2.4, 1.4–4.0)	20/316 (6.3, 3.9–9.6)	4/96 (4.2, 1.1–10.3)	1/39 (2.6, 0.06–13.5)	12, 26
Santa Clara County	0/6 (0, 0–45.9)	0/6 (0, 0–45.9)	0/21 (0, 0–16.1)	0/21 (0, 0–16.1)	This study
	2/98 (2.0, 0.2–7.2)	2/98 (2.0, 0.2–7.2)	4/75 (5.3, 1.5–13.1)	5/75 (6.7, 2.2–14.9)	17^*b*^
	3/182 (1.6, 0.3–4.7)	0/167 (0, 0–2.2)	9/134 (6.7, 3.1–12.4)	1/39 (2.6, 0.06–13.5)	12, 26
Santa Cruz	12/299 (4.0, 2.1–6.9)	4/299 (1.3, 0.4–3.4)	0/33 (0, 0–10.6)	0/33 (0, 0–10.6)	This study
	3/893 (0.3, 0.1–1.0)	7/752 (0.9, 0.4–1.9)	16/476 (3.4, 1.9–5.4)	8/443 (1.8, 0.8–3.5)	12, 26
Sonoma	7/253 (2.8, 1.1–5.6)	5/253 (2.0, 0.6–4.6)	1/80 (1.3, 0.03–6.8	2/80 (2.5, 0.3–8.7)	This study
	1/216 (0.5, 0.01–2.6)	0/53 (0, 0–6.7)	3/337 (0.9, 0.2–2.6)	1/159 (0.6, 0.02–3.5)	12, 26
Region-wide (Marin, Mendocino, Monterey, Napa, Santa Clara, Santa Cruz, Sonoma counties)	70/2,386 (2.9, 2.3–3.7) 24/2,459 (1.0, 0.6–1.4)	30/2,386 (1.3, 0.8–1.8) 8/1,482 (0.5, 0.2–1.1)	18/567 (3.2, 1.9–5.0) 55/1,674 (3.3, 2.5–4.3)	29/567 (5.1, 3.5–7.3) 13/1,001 (1.3, 0.7–2.2)	This study 12, 26
State-wide	37/6,036 (0.6, 0.5–1.0)	51/6,036 (0.8, 0.6–1.1)	70/2,188 (3.2, 2.5–4.0)	30/2,188 (1.4, 0.9–2.0)	12, 26

a Prevalence data are presented as number positive/number tested (percentage positive, 95% confidence interval).

b Data obtained from sites where *Borrelia* were identified to the species level, or where zero ticks were infected.

**TABLE 3:** Study sites, habitat types, and prevalence of Borrelia burgdorferi *sensu lato*, B. miyamotoi, and Anaplasma phagocytophilum in questing adult and nymphal western black-legged ticks (Ixodes pacificus)^*a*^

Site (collection date)	Habitat type	Prevalence^*b*^					
		Adult ticks			Nymphal ticks		
		B. burgdorferi *sensu lato*	B. miyamotoi	A. phagocytophilum	B. burgdorferi *sensu lato*	B. miyamotoi	A. phagocytophilum
Marin Co.							
Bolinas Lagoon (May 2018)	Woodland				6/90 (6.7, 2.5–13.9); 2 Bb ss	16/90 (17.8, 10.5–27.3); 5 Bm	7/90^Bb sl coinf^ (7.8, 3.2–15.4)
Cascade Canyon OSP (May 2016)	Woodland				1/34 (2.9, 0.1–15.3); 1 Bb ss	0/34 (0, 0–10.3)	0/34 (0, 0–10.3)
China Camp SP (Jan and May 2016)	Woodland	1/89 (1.1, 0.03–6.1); 1 Bb ss	3/89 (3.4, 0.7–9.5); 2 Bm	2/89 (2.2, 0.3–7.9)	0/67 (0, 0–5.4)	5/67 (7.5, 2.5–16.6); 2 Bm	2/67^Bm coinf^ (3.0, 0.4–10.4)
Lucas Valley (Jan and May 2016)	Grassland	0/42 (0, 0–8.4)	0/42 (0, 0–8.4)	0/42 (0, 0–8.4)	0/1 (0, 0–97.5)	1/1 (100, 2.5–100)	0/1 (0, 0–97.5)
Lucas Valley (Jan and May 2016)	Woodland	0/130 (0, 0–2.8)	1/130 (0.8, 0.02–4.2); 1 Bm	0/130 (0, 0–2.8)	0/14 (0, 0–23.2)	0/14 (0, 0–23.2)	0/14 (0, 0–23.2)
Marin Headlands—Owl Trail (Jan 2016)	Coastal chaparral	10/171 (5.8, 2.8–10.5); 5 Bb ss, 2 B am	1/171 (0.6, 0.01–3.2)	2/171 (1.2, 0.1–4.1)			
Northern Marin (May 2016)	Woodland				0/63 (0, 0–5.7)	2/63 (3.2, 0.4–11.0)	0/63 (0, 0–5.7)
Olompali SP (Jan and May 2016, May 2017)	Woodland	26/330 (7.9, 5.2–11.3); 3 Bb ss	10/330 (3.0, 1.5–5.5); 5 Bm	1/325 (0.3, 0.01–1.7)	6/107 (5.6, 2.1–11.8); 6 Bb ss	8/107 (7.5, 3.3–14.2); 5 Bm	1/76 (1.3, 0.03–7.1)
Point Reyes National Seashore—Five Brooks (May 2016)	Woodland				0/4 (0, 0–60.2)	1/4 (25, 0.6–80.6)	0/4 (0, 0–60.2)
Point Reyes National Seashore—McClure Beach (Jan and May 2016)	Coastal chaparral	2/100 (2.0, 0.2–7.0); 1 Bb ss	0/100 (0, 0–3.6)	0/100 (0, 0–3.6)			
Point Reyes National Seashore—Tomales Point (Jan 2016)	Coastal prairie, coastal chaparral	0/35 (0, 0–10.0)	0/35 (0, 0–10.0)	0/35 (0, 0–10.0)			
Samuel P. Taylor SP (Jan and May 2016)	Woodland	0/82 (0, 0–4.4)	2/82 (2.4, 0.3–8.5)	0/82 (0, 0–4.4)	2/90 (2.2, 0.3–7.8); 1 Bb ss	0/90 (0, 0–4.0)	0/90 (0, 0–4.0)
Tamalpais SP (Jan 2016)	Chaparral	0/15 (0, 0–21.8)	0/15 (0, 0–21.8)	0/15 (0, 0–21.8)			
Tennessee Valley (Jan 2016)	Coastal chaparral	3/39 (7.7, 1.6–20.9); 1 Bb ss; 1 B am	0/39 (0, 0–9.0)	0/39 (0, 0–9.0)			
Tennessee Valley (Jan and May 2016)	Woodland	0/6 (0, 0–45.9)	0/6 (0, 0–45.9)	0/6 (0, 0–45.9)	2/13 (15.4, 1.9–45.4)	0/13 (0, 0–24.7)	0/13 (0, 0–24.7)
Mendocino Co.							
Glass Beach (Jan 2018)	Coastal prairie, coastal chaparral	0/13 (0, 0–24.7)	0/13 (0, 0–24.7)	0/13 (0, 0–24.7)			
Hendy Woods SP (Dec 2017)	Woodland	0/7 (0, 0–41.0)	0/7 (0, 0–41.0)	0/7 (0, 0–41.0)			
Mendocino Headland (Jan 2018)	Coastal prairie, coastal chaparral	0/3 (0, 0–70.8)	0/3 (0, 0–70.8)	0/3 (0, 0–70.8)			
Monterey Co.							
Andrew Molera SP (Jan 2018)	Woodland	1/149 (0.7, 0.02–3.7); 1 Bbis	0/149 (0, 0–2.4)	2/149 (1.3, 0.2–4.8)			
Elkhorn Slough National Estuarine Research Reserve (Dec 2015 and May 2016)	Coastal chaparral, coastal prairie, woodland	0/72 (0, 0–5.0)	0/72 (0, 0–5.0)	0/72 (0, 0–5.0)	0/1 (0, 0–97.5)	0/1 (0, 0–97.5)	
Garrapata SP (Dec 2015)	Coastal chaparral	0/155 (0, 0–2.4)	0/155 (0, 0–2.4)	0/155 (0, 0–2.4)			
Garland Ranch Regional Park (Dec 2015 and May 2016)	Woodland	0/70 (0, 0–5.1)	2/70 (2.8, 0.3–10.0); 1 Bm	0/70 (0, 0–5.1)			
Garland Ranch Regional Park—Garzas Creek (Dec 2015)	Woodland	0/53 (0, 0–6.7)	0/53 (0, 0–6.7)	0/53 (0, 0–6.7)			
Pfeiffer Big Sur SP (May 2016)	Woodland	3/46 (6.5, 1.4–17.9); 2 Bb ss	1/46 (2.2, 0.1–11.5); 1 Bm	0/46 (0, 0–7.7)			
Point Lobos SP (Jan 2016)	Coastal prairie	0/28 (0, 0–12.3)	2/28 (6.7, 0.8–22.1); 2 Bm	0/28 (0, 0–12.3)			
Prewitt Loop Trail (Dec 2015)	Coastal chaparral	1/55 (1.8, 0.05–9.7)	0/55 (0, 0–6.5)	0/55 (0, 0–6.5)			
Salmon Creek Falls (Dec 2015, Jan and May 2016)	Woodland	0/57 (0, 0–6.3)	0/57 (0, 0–6.3)	1/57 (1.8, 0.04–9.4)			
Sand Dollar Beach (Dec 2015)	Coastal chaparral	0/3 (0, 0–70.8)	0/3 (0, 0–70.8)	0/3 (0, 0–70.8)			
Toro Park (Dec 2015)	Woodland	0/5 (0, 0–52.2)	0/5 (0, 0–52.2)	0/5 (0, 0–52.2)			
Napa Co.							
Robert Louis Stevenson SP (May and Dec 2017)	Woodland	3/71 (4.2, 0.9–11.9); 3 Bb ss	1/71 (1.4, 0.4–7.6); 1 Bm	0/57 (0, 0–6.3)	0/11 (0, 0–28.5)	0/11 (0, 0–28.5)	
Skyline Wilderness Park (May 2017)	Woodland	0/2 (0, 0–84.2)	0/2 (0, 0–84.2)		0/9 (0, 0–33.6)	0/9 (0, 0–33.6)	
Santa Clara Co.							
Sanborn Creek (May 2017)	Woodland	0/3 (0, 0–70.8)	0/3 (0, 0–70.8)		0/3 (0, 0–70.8)	0/3 (0, 0–70.8)	
Skyline Boulevard (May 2017)	Woodland	0/3 (0, 0–70.8)	0/3 (0, 0–70.8)		0/18 (0, 0–18.5)	0/18 (0, 0–18.5)	
Santa Cruz Co.							
Big Basin Highway (May 2017)	Woodland	0/4 (0, 0–60.2)	0/4 (0, 0–60.2)		0/3 (0, 0–70.8)	0/3 (0, 0–70.8)	
Big Basin SP (Dec 2016)	Redwood forest/meadow	1/28 (3.6, 0.1–18.3); 1 Bb ss	1/28 (3.6, 0.1–18.3); 1 Bm	0/28 (0, 0–12.3)			
Big Basin SP— Rancho del Oso (Dec 2016 and May 2017)	Woodland	4/73 (5.5, 1.5–13.4); 4 Bb ss	0/73 (0, 0–4.9)		0/15 (0, 0–21.8)	0/15 (0, 0–21.8)	
Forest of Nisene Marks SP (Dec 2016 and May 2017)	Redwood forest, woodland	2/20 (10.0, 1.2–31.7); 1 Bb ss	0/20 (0, 0–16.8)	0/6 (0, 0–45.9)	0/4 (0, 0–60.2)	0/4 (0, 0–60.2)	
Henry Cowell SP (Dec 2016)	Redwood forest	1/38 (2.6, 0.1–13.8); 1 Bb ss	0/38 (0, 0–9.3)				
Larkin Valley (Jan and May 2017)	Woodland	0/20 (0, 0–16.8)	0/20 (0, 0–16.8)	0/20 (0, 0–16.8)	0/11 (0, 0–28.5)	0/11 (0, 0–28.5)	
Waddell Creek (Dec 2016)	Woodland	3/42 (7.1, 1.5–19.5); 2 Bb ss	0/42 (0, 0–8.4)	0/42 (0, 0–8.4)			
Wilder Ranch SP (Dec 2016)	Woodland/coastal chaparral	1/74 (1.4, 0.03–7.3)	3/74 (4.1, 0.8–11.4); 2 Bm				
Sonoma Co.							
Austin Creek (May and Dec 2017)	Woodland	2/76 (2.6, 0.3–9.2); 2 Bb ss	3/76 (3.9, 0.8–11.1); 3 Bm	0/68 (0, 0–5.3)	1/36 (2.8, 0.1–14.5)	1/36 (2.8, 0.1–14.5); 1 Bm	
Goat Rock (Dec 2017)	Coastal chaparral	0/38 (0, 0–9.3)	1/38 (2.6, 0.1–13.8); 1 Bm	0/38 (0, 0–9.3)			
Healdsburg Ridge OSP (May and Dec 2017)	Woodland	1/64 (1.6, 0.04–8.4); 1 Bb ss	0/64 (0, 0–5.6)	0/60 (0, 0–6.0)	0/39 (0, 0–9.0)	1/39 (2.6, 0.1–13.5)	
Pomo Canyon (Jan 2017)	Grassland/coastal chaparral	0/34 (0, 0–10.3)	0/34 (0, 0–10.3)	0/34 (0, 0–10.3)			
Salt Point SP (Jan 2018)	Coastal prairie	2/32 (6.25, 0.7–20.8); 2 Bb ss	0/32 (0, 0–10.9)	1/32^Bb ss coinf^ (3.1, 0.1–16.2)			
Trione-Annadel SP (Dec 2017)	Woodland	2/9 (22.2, 6.0–60.0); 2 Bb ss	0/9 (0, 0–33.6)	0/9 (0, 0–33.6)			

a Abbreviations: Bb sl, B. burgdorferi *sensu lato*; Bb ss, B. burgdorferi *sensu stricto*; Bm, B. miyamotoi; Bbis, *B. bissettiae*; Bam, *B. americana*; OSP, Open Space Preserve; SP, State Park.

b Prevalence data are presented as number positive/number tested (percentage positive, 95% confidence interval). Superscripts indicate coinfections and represent one coinfected tick in the total sample. Sequenced samples are described following the reported prevalence, e.g., 6/90 ticks positive for Borrelia burgdorferi *sensu lato*, 2 of which were sequenced as B. burgdorferi *sensu stricto*. Only a subset of positive samples were sequenced.

Several individual sites exhibited B. burgdorferi *sensu lato* prevalence greater than 3.7% in adult ticks, i.e., higher than the confidence intervals generated when all the data were aggregated (2.3 to 3.7%). Some of these sites included different species of B. burgdorferi *sensu lato*, such as B. burgdorferi *sensu stricto*, *B. americana*, and *B. bissettiae* (e.g., Marin Headlands and Tennessee Valley), and some were in habitats not traditionally associated with B. burgdorferi *sensu lato* (e.g., chaparral or redwood forest). We did not observe B. burgdorferi *sensu lato* in adult I. pacificus ticks at 11/27 of the sites at which we tested >30 ticks (11 sites representing 768 ticks).

### B. miyamotoi prevalence in adult ticks.

At the regional level (all counties combined), B. miyamotoi in adult ticks occurred at a lower prevalence than B. burgdorferi *sensu lato*: 1.3% (95% CI = 0.8 to 1.8%) (Table 2). When county-level data were compared, B. miyamotoi and B. burgdorferi *sensu lato* were observed at comparable prevalences in Monterey (0.7% for both species) and Sonoma (2.8% B. burgdorferi *sensu lato*; 2.0% B. miyamotoi) counties.

At individual sites where B. miyamotoi was present (and where samples sizes were >30; *n* = 9), the prevalence of B. miyamotoi often exceeded the prevalence defined by the confidence intervals generated by the aggregated data (0.8 to 1.8%), ranging from 2.2 to 3.9% at 6 sites (Tables 2 and 3). Like B. burgdorferi *sensu lato*, B. miyamotoi was not observed in Mendocino or Santa Clara counties, where samples were small.

### B. burgdorferi *sensu lato* prevalence in nymphal ticks.

In total, B. burgdorferi *sensu lato* prevalence in nymphal western black-legged ticks was 3.2% (95% CI = 1.9 to 5.0%) (Table 2). The vast majority of *Borrelia*-positive nymphal ticks were collected in Marin County: 17/18 B. burgdorferi *sensu lato*-positive nymphs and 27/29 B. miyamotoi*-*positive nymphs were observed in Marin County (496 nymphs were collected in Marin County; a total of 155 nymphs were collected from other counties). All the B. burgdorferi *sensu lato* samples sequenced from nymphs were determined to be B. burgdorferi *sensu stricto* (10/10).

At individual sites, nymphal infection prevalence was occasionally higher than the combined prevalence, e.g., 6.7% at Bolinas Lagoon (95% CI = 2.5 to 13.9%; *n* = 90) and 5.6% at Olompali State Park (95% CI = 2.1 to 11.8%; *n* = 107).

### B. miyamotoi prevalence in nymphal ticks.

In contrast to the adult stage, B. miyamotoi was observed in higher prevalence than B. burgdorferi in nymphal ticks at the regional level: B. miyamotoi prevalence was 5.1% (95% CI = 3.5 to 7.3%) (Table 2), though there was no statistical difference (B. burgdorferi *sensu lato* versus B. miyamotoi; Fisher exact test *P* = 0.14; chi-square *P* = 0.10).

Nymphal infection prevalence of B. miyamotoi reached 17.8% (95% CI = 10.5 to 27.3; *n* = 90) in Bolinas Lagoon and was also high in China Camp (7.5%; 95% CI = 2.5 to 16.6; *n* = 67) and Olompali state parks (7.5%; 95% CI = 3.3 to 14.2; *n* = 107) (Table 3).

### B. miyamotoi prevalence in larval ticks.

A pooled sample of two larvae, collected at Olompali State Park, Marin County, tested positive for B. miyamotoi. All other larvae (*n* = 85) were negative for B. miyamotoi, and these were also collected in Marin County: Olompali State Park (*n* = 22), China Camp State Park (*n* = 16), Cascade Canyon Open Space (*n* = 35), Northern Marin (*n* = 9), Bolinas Lagoon (*n* = 2), and Samuel P. Taylor State Park (*n* = 1). B. miyamotoi prevalence in I. pacificus larvae was therefore minimally 4.2% (1/24; 95% CI = 0.1 to 21.1%) in Olompali State Park, assuming just a single infected larva in the tested pool, and 1.1% (1/87; 95% CI = 0.03 to 6.2%) overall in Marin County.

At Olompali State Park, where we observed B. miyamotoi in all the tick life stages, prevalence was 4.2% in larvae (1/24), 7.5% in nymphs (8/107), and 3.0% in adults (10/330). B. miyamotoi prevalence was not statistically different across life stages (Fisher’s exact test, *P* = 0.12). Comparing B. miyamotoi prevalence in larvae from May 2016 (1/24) with nymphs in May 2017 (0/31), i.e., the same tick cohort, there was also no statistical difference in prevalence (Fisher’s exact test, *P* = 0.44). Aggregating data from across Marin County, B. miyamotoi prevalence was 1.1% in larvae (1/87; 95% CI = 0.03 to 6.2%), 6.8% in nymphs (33/483; 95% CI = 4.7 to 9.5%), and 1.6% in adults (17/1,039; 95% CI = 1.0 to 2.6%), revealing a significant difference in prevalence between life stages (Fisher’s exact test, *P* < 0.001).

### *B. americana* and *B. bissettiae*.

*B. americana* was observed in three I. pacificus ticks, all collected in coastal chaparral habitat in Marin County. *B. americana* constituted 2/7 (28.6%) of the sequenced B. burgdorferi *sensu lato* samples from adult ticks on the Owl Trail, Marin Headlands, and 1/2 (50%) of the sequenced B. burgdorferi *sensu lato* samples from adult ticks in nearby Tennessee Valley. At both sites, both *B. americana* and B. burgdorferi *sensu stricto* were observed in the adult tick populations, and B. miyamotoi was also observed at Owl Trail.

*B. bissettiae* was observed just once, in an adult tick from Andrew Molera State Park in Monterey County, where it accounted for the only *Borrelia*-positive result.

### Anaplasma phagocytophilum prevalence and coinfections.

Anaplasma phagocytophilum was observed in Marin County at prevalence up to 7.8% (95% CI = 3.2 to 15.4%; *n* = 90; Bolinas Lagoon), though its presence was sporadic (4/14 sites), as well as in Monterey County (2/11 sites) and in Sonoma County (1/6 sites).

We observed three coinfected ticks: one adult tick from Salt Point State Park with B. burgdorferi *sensu stricto* (sequenced) and A. phagocytophilum, one nymph with a similar microbial combination (B. burgdorferi *sensu lato*—not sequenced) from Bolinas Lagoon, and one nymph with B. miyamotoi (sequenced) and A. phagocytophilum from China Camp State Park.

### Coexisting tick-borne pathogens.

**FIG 4:**
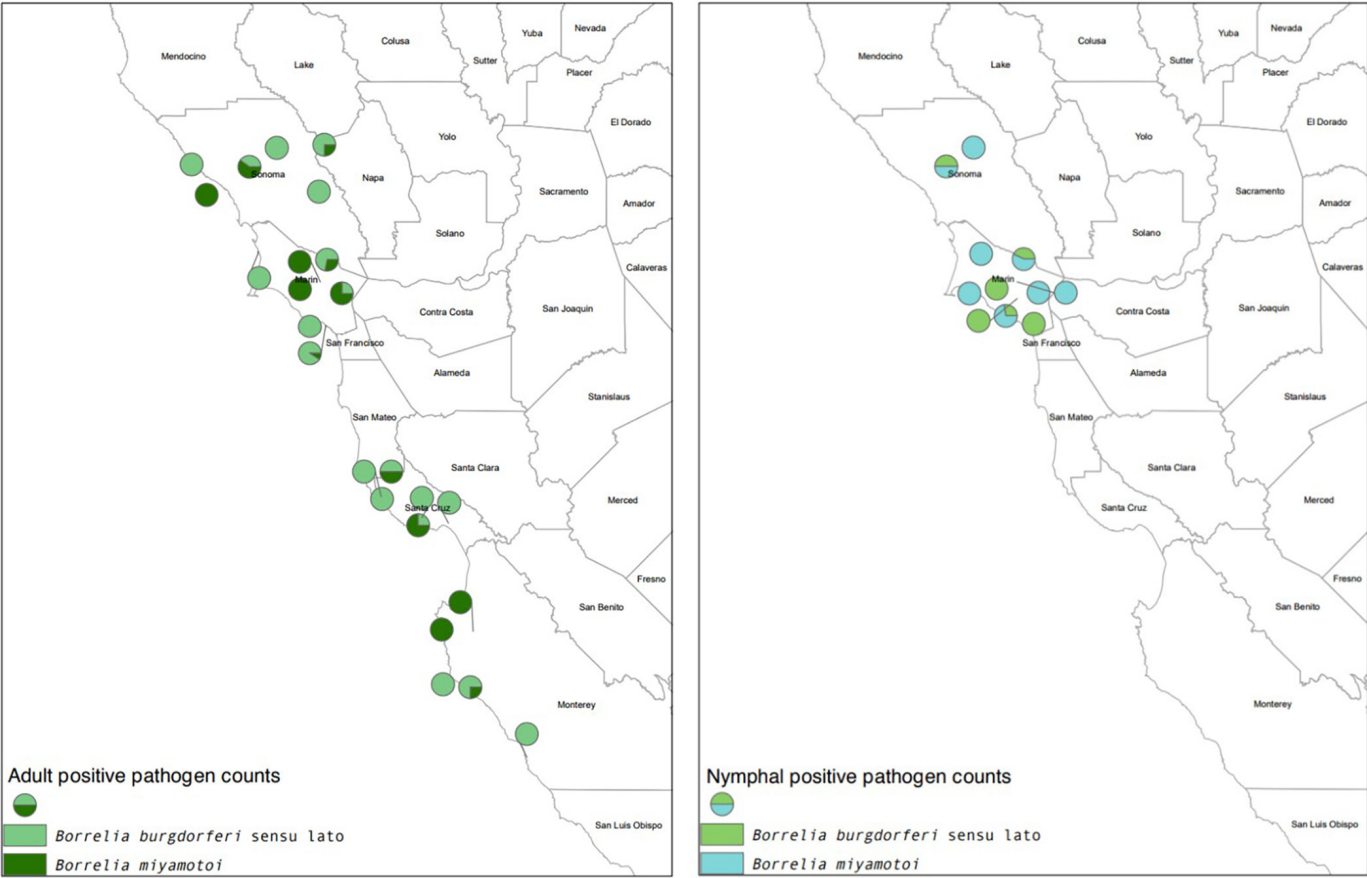
Maps showing proportional counts of positive samples from study sites for Borrelia burgdorferi *sensu lato* and B. miyamotoi in nymphal (left) and adult (right) western black-legged ticks (Ixodes pacificus). The maps were created in ArcMap, and the polygon feature class of the California county boundaries was downloaded from ArcGIS (credits: U.S. Bureau of Reclamation, California Department of Conservation, California Department of Fish and Game, California Department of Forestry and Fire Protection, and National Oceanic and Atmospheric Administration).

There was no obvious pattern to coexistence of B. burgdorferi *sensu lato* and B. miyamotoi (Fig. 4). At sites where *Borrelia* species were observed, 13 sites had only B. burgdorferi *sensu lato*, six sites had only B. miyamotoi, and the two species coexisted at 10 sites.

At the site level, B. burgdorferi *sensu lato* was sometimes present in adult tick samples but absent from nymphs (e.g., China Camp State Park). The contrasting pattern (infected nymphs and uninfected adults) also appeared (e.g., Samuel P. Taylor State Park). Similarly, B. miyamotoi was observed in adult ticks at Samuel P. Taylor State Park but was not found in the collected nymphal ticks.

### Habitat associations.

*Borrelia-*positive ticks were observed in coastal chaparral and prairie habitats in Sonoma, Marin, Santa Cruz, and Monterey counties. Species identified by sequencing in these habitats represented the full gamut of *Borrelia* species identified in this study: B. burgdorferi *sensu stricto*, B. miyamotoi, *B. americana*, and *B. bissettiae* (Table 3; Fig. 3).

**FIG 5:**
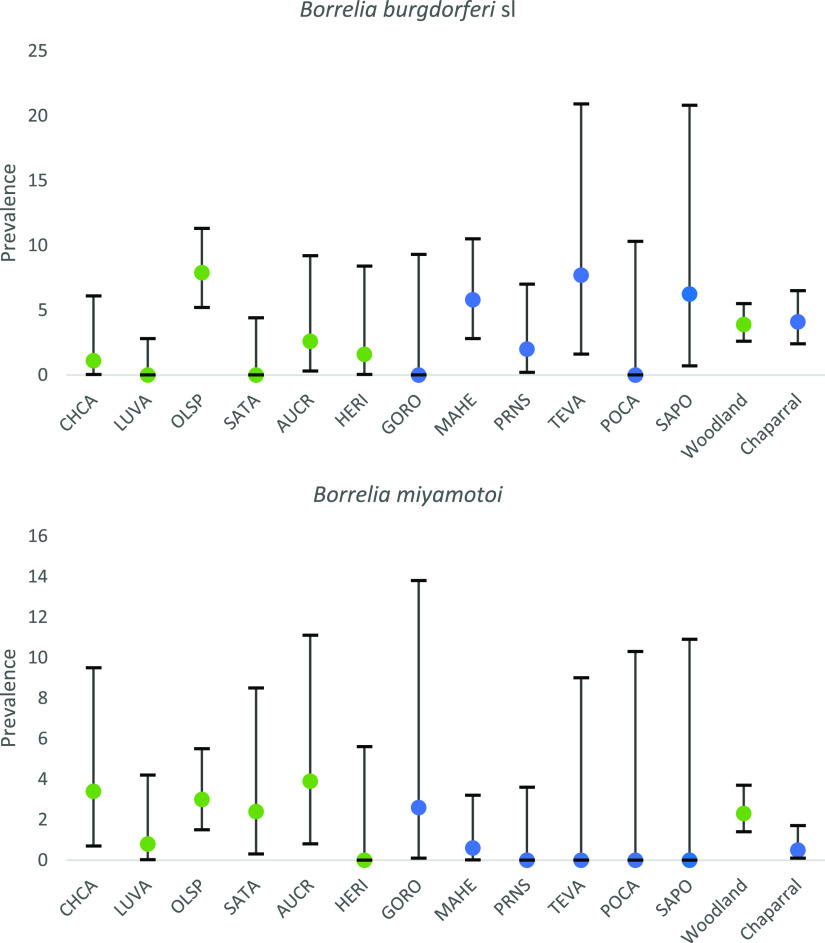
Comparisons of the prevalence of Borrelia burgdorferi *sensu lato* (sl) and Borrelia miyamotoi in adult Ixodes pacificus in woodland (green) and chaparral (blue) habitats of Marin and Sonoma counties, California. Bars show 95% confidence intervals for several sites; *n* > 30 for all sites. Sites were in Marin County (CHCA, China Camp State Park; LUVA, Lucas Valley woodland; OLSP, Olompali State Park; SATA, Samuel P. Taylor State Park; MAHE, Marin Headlands; PRNS, Point Reyes National Seashore [McClure Beach]; TEVA, Tennessee Valley [chaparral]) and Sonoma County (AUCR, Austin Creek; HERI, Healdsburg Ridge; GORO, Goat Rock; POCA, Pomo Canyon; SAPO, Salt Point).

We compared tick-borne-pathogen prevalence in woodland and coastal chaparral habitat for our sample sites in Marin and Sonoma counties, using adult tick populations where *n* was >30. We restricted analyses to these two counties because they are coastal and the two habitats were well represented (*n* = 6 for woodland and chaparral sites), and we used just adult ticks because nymphs are difficult to collect in chaparral. There was no significant difference in the prevalence of B. burgdorferi *sensu lato* in the two habitats after aggregation of the data (χ^2^ = 0.03; *P* = 0.86), and site-level prevalence was similar (Fig. 5). In contrast, B. miyamotoi prevalence was higher in woodland habitats (χ^2^ = 5.57; *P* = 0.018) (Fig. 5). Prevalence of A. phagocytophilum did not differ according to habitat, though prevalence was low across sites (Fisher's exact test, *P* = 0.67).

### Comparisons to previous reports.

Our regional estimates of B. miyamotoi prevalence in I. pacificus ticks were 1.3% in adult ticks (95% CI, 0.8 to 1.8%), and 5.1% (95% CI, 3.5 to 7.3%) in nymphal ticks; compared to prior reports of 0.5% (95% CI, 0.2 to 1.1%) and 1.3% (95% CI, 0.7 to 2.2%) in adult and nymphal ticks, respectively, from these same counties, and statewide estimates of 0.8% (95% CI, 0.6 to 1.1%) and 1.4% (95% CI, 0.9 to 2.0%) (12) (Table 2). Prevalence of B. miyamotoi in adult ticks was not quite statistically significantly different from the regional aggregation and statewide levels reported by Padgett et al. (12) (Fisher’s exact test, *P* = 0.058). However, our nymphal B. miyamotoi infection prevalence was significantly higher (Fisher’s exact test, *P* < 0.001).

For B. burgdorferi *sensu lato*, our regional estimates of prevalence in I. pacificus ticks were 2.9% (95% CI, 2.3 to 3.7%) in adult ticks and 3.2% in nymphal ticks (95% CI, 1.9 to 5.0%), compared to prior reports of 1.0% (95% CI, 0.6 to 1.4%) in adults ticks and 3.3% (95% CI, 2.5 to 4.3%) in nymphal ticks from these same counties and statewide estimates of 0.6% (95% CI, 0.5 to 1.0%) and 3.2% (95% CI, 2.5 to 4.0%) in adult and nymphal ticks, respectively (12, 26). Our study observed a higher B. burgdorferi *sensu lato* prevalence in adult ticks than the prior studies (Fisher’s exact test, *P* < 0.001), but there was no difference in nymphal B. burgdorferi *sensu lato* infection prevalence (Fisher’s exact test, *P* = 0.99).

## DISCUSSION

### Describing infection prevalence.

The tick-borne pathogens Borrelia burgdorferi *sensu lato*, B. miyamotoi, and Anaplasma phagocytophilum were observed sporadically in questing tick populations across northern California. Aggregating across the region, we found higher infection prevalence of B. miyamotoi in nymphal ticks and higher B. burgdorferi *sensu lato* infection prevalence in adult ticks than reported in recent studies (12, 26). Because the sampled ticks were collected at different times, these differences in prevalence may reflect trends in *Borrelia* infection patterns, interannual fluctuations in prevalence, or simply variation due to chance. Nevertheless, multiple measures of tick-borne infection prevalence are useful to gain a broader picture of local and regional pathogen prevalence, rather than relying on a single data source.

**FIG 6:**
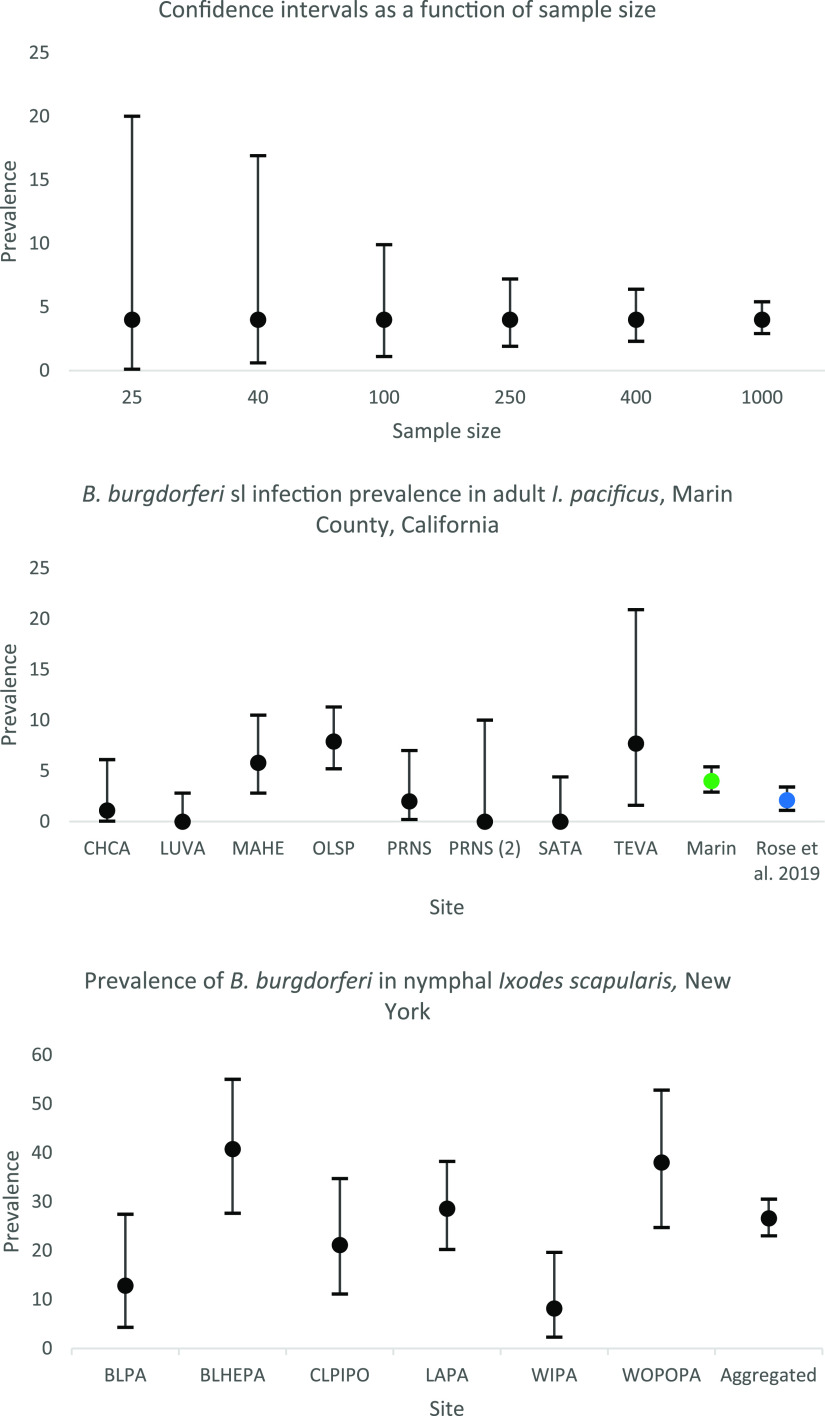
(Top) Patterns of 95% confidence intervals as a function of growing sample size (range, 25 to 1,000) for a set prevalence of 4%. (Middle) Prevalence (95% CI) of B. burgdorferi *sensu lato* in adult western black-legged ticks (I. pacificus) from sites in Marin County (sample sizes > 30), as well as aggregated prevalence for the entire county (green, this study; blue, reference 26) (CHCA, China Camp State Park; LUVA, Lucas Valley woodland; MAHE, Marin Headlands; OLSP, Olompali State Park; PRNS, Point Reyes National Seashore [McClure Beach]; PRNS (2), Point Reyes National Seashore [Tomales Point]; SATA, Samuel P. Taylor State Park; TEVA, Tennessee Valley [chaparral]). (Bottom) Prevalence (95% CI) of B. burgdorferi in black-legged ticks (I. scapularis) from a subset of sites in New York as well as aggregated prevalence across all sites (53) (BLPA, Bloomingdale Park; BLHEPA, Blue Heron Park; CLPIPO, Clay Pit Ponds; WIPA, Willowbrook Park; WOPOPA, Wolfe’s Pond Park).

A key component and rationale of surveillance of ticks and tick-borne pathogens in the wild is to be able to represent the risk of exposure to humans. Often the data are summarized across the largest spatial extent. So, for example, despite sampling at multiple sites, data are reported as “In total, *x*% of ticks were infected with B. burgdorferi…,” a behavior we have been guilty of (e.g., see references 17 and 29). However, aggregation of data into a single statistic can underrepresent the risk of exposure to tick-borne pathogens at sites where prevalence is higher, in part because the portrayed prevalence is deflated by including data from sites where tick abundance and/or pathogen prevalence is low. As an illustration, B. burgdorferi *sensu stricto* is known to be extremely rare in southern California—documented in only a single I. pacificus tick, from 5,571 ticks screened during three different studies (0.02%; 95% CI = 0.0005 to 0.1) (26, 27, 30). Consequently, describing B. burgdorferi prevalence in ticks for the state of California could dramatically underrepresent Lyme disease risk for northern Californians if all data are aggregated. This phenomenon can occur at a smaller scale: for example, B. miyamotoi prevalence in nymphal I. pacificus in Bolinas Lagoon, Marin County, was measured as 17.8%, with a 95% CI of 10.5 to 27.3% and a decent sample size of 90 nymphs. However, when aggregated across the county, nymphal infection prevalence of B. miyamotoi falls to 6.6% (95% CI = 4.4 to 9.4%), and in the Bay Area region, it slips to 5.1% (95% CI = 3.5 to 7.3) (Fig. 6; Table 3). Counties are often used as the spatial unit for reporting vector-borne and other disease metrics, but doing so can obfuscate smaller-scale patterns of disease risk (31) or result in erroneous interpretations of disease drivers (32). One solution to portray disease prevalence is to portray the 95% confidence intervals, which inherently demonstrate the range of interpretable prevalence. However, this method also has shortfalls if samples are aggregated, as the confidence intervals shrink with increasing sample size (Fig. 6), suggesting improved confidence but ignoring the fact that the source data are combined from multiple sites.

We advocate for transparently sharing data from all sites so that scientists, concerned citizens, physicians, public health agencies, and vector control districts can make appropriate judgments regarding the relevant risk of tick-borne disease. For example, it is important to understand that outdoor recreation in southern California has a lower risk for tick-borne disease exposure than outdoor recreation in northern California. These nuances can be important for treatment, control, and educational opportunities. In addition, zoonotic disease systems often exhibit fine-scale spatial patterns, and sharing these data at the site level may help future studies examining disease ecology and environmental drivers (33). Similarly, prevalence patterns of *Borrelia* likely will vary across time even at the same sites (34, 35).

Typically, infection prevalence is reported for a single pathogen, e.g., prevalence of B. miyamotoi in a tick population or sample. This method of data presentation fails to recognize the fact that a population of ticks can often harbor multiple pathogens (35) and that reporting on a single pathogen species underestimates local risk of tick-borne disease. To provide an example, for the same Bolinas Lagoon tick population, pathogen prevalence is 6.7% for B. burgdorferi *sensu lato*, 17.8% for B. miyamotoi, and 7.8% for A. phagocytophilum. The overall prevalence of ticks with human pathogens in this population is 31.1% (28/90, as one tick was coinfected; 95% CI = 21.8 to 41.7%). The difference when multiple pathogens are considered is not always so pronounced; e.g., cumulative tick-borne-pathogen prevalence in China Camp State Park is 9.0% (6/61; 95% CI = 3.4 to 18.5%), compared to 7.5% for B. miyamotoi and 3.0% for A. phagocytophilum (one tick was coinfected). However, it is important to consider multiple pathogens when assessing local disease risk.

### Vertical transmission of B. miyamotoi.

B. miyamotoi is known to be vertically transmitted in Ixodes scapularis (18, 19), has been observed in I. pacificus larvae (36), and is able to infect small mammals that ticks feed on (18, 25, 36, 37). It is unclear whether infection dynamics in natural populations require amplification by horizontal transmission from the vertebrate hosts.

Recently, data from the Bay Area were used to argue that horizontal transmission is required for B. miyamotoi transmission in California, based on an increase in infection prevalence across developing tick life stages (36). However, this pattern was generated from data that included a single infected larva and aggregation of infection prevalence in tick life stages from eight different sites spanning five counties. At the site where the B. miyamotoi*-*infected larva was observed (Heinz Open Space, Santa Clara County), infection prevalence was 0.5% (1/201) in larvae, 0% in nymphs (0/19), and 0% (0/1) in adults (Fisher’s exact test, *P* = 1.0). At sites with higher B. miyamotoi prevalence, e.g., Windy Hill, San Mateo County, there were significant differences between the life stages (Fisher’s exact test, *P* = 0.01), though this statistical difference is driven by the lack of observed infected larvae (0/58 larvae, 5/57 nymphs, 16/137 adults; Fisher’s exact test for only nymph and adult stages, *P* = 0.62).

In our study, at Olompali State Park, B. miyamotoi was observed in all I. pacificus life stages, though in only a single larva, and there was no significant change in infection prevalence. Because we also identified only a single B. miyamotoi*-i*nfected larva, interpretations of both studies on B. miyamotoi transmission across tick life stages should be viewed with caution. Though we suspect that small mammals do indeed play a role in B. miyamotoi infection dynamics, there are not yet enough field data from the California system to support this hypothesis. Increased surveillance for B. miyamotoi in larval I. pacificus and experimental tests of reservoir competence for B. miyamotoi in vertebrate hosts are required to demonstrate that horizontal transmission is important in the California system (25).

### *Borrelia* ecology and habitat type.

We observed a diversity of *Borrelia* species in coastal habitats. Coastal prairie and coastal chaparral have received relatively little attention compared to woodland habitats in northern California (e.g., see references 3, 17, and 38), and at first glance these habitats would appear to be low risk for *Borrelia* exposure due to the lack of recognized mammalian reservoir hosts; e.g., western gray squirrels are not common in these habitats. However, the prevalence of B. burgdorferi *sensu lato* in adult ticks in coastal chaparral in Marin and Sonoma counties was equivalent to that in woodlands, suggesting that this habitat may pose a risk for Lyme borreliosis exposure when adult tick populations are abundant in the winter.

Nymphal I. pacificus ticks were not collected in the coastal grass- or shrublands. We suspect that they are present but that tick flagging is not an effective way to collect this life stage in chaparral or grassland. Future investigations should attempt to survey tick hosts, e.g., western fence lizards, to examine the ecology of nymphal I. pacificus in these habitats (30).

**FIG 7:**
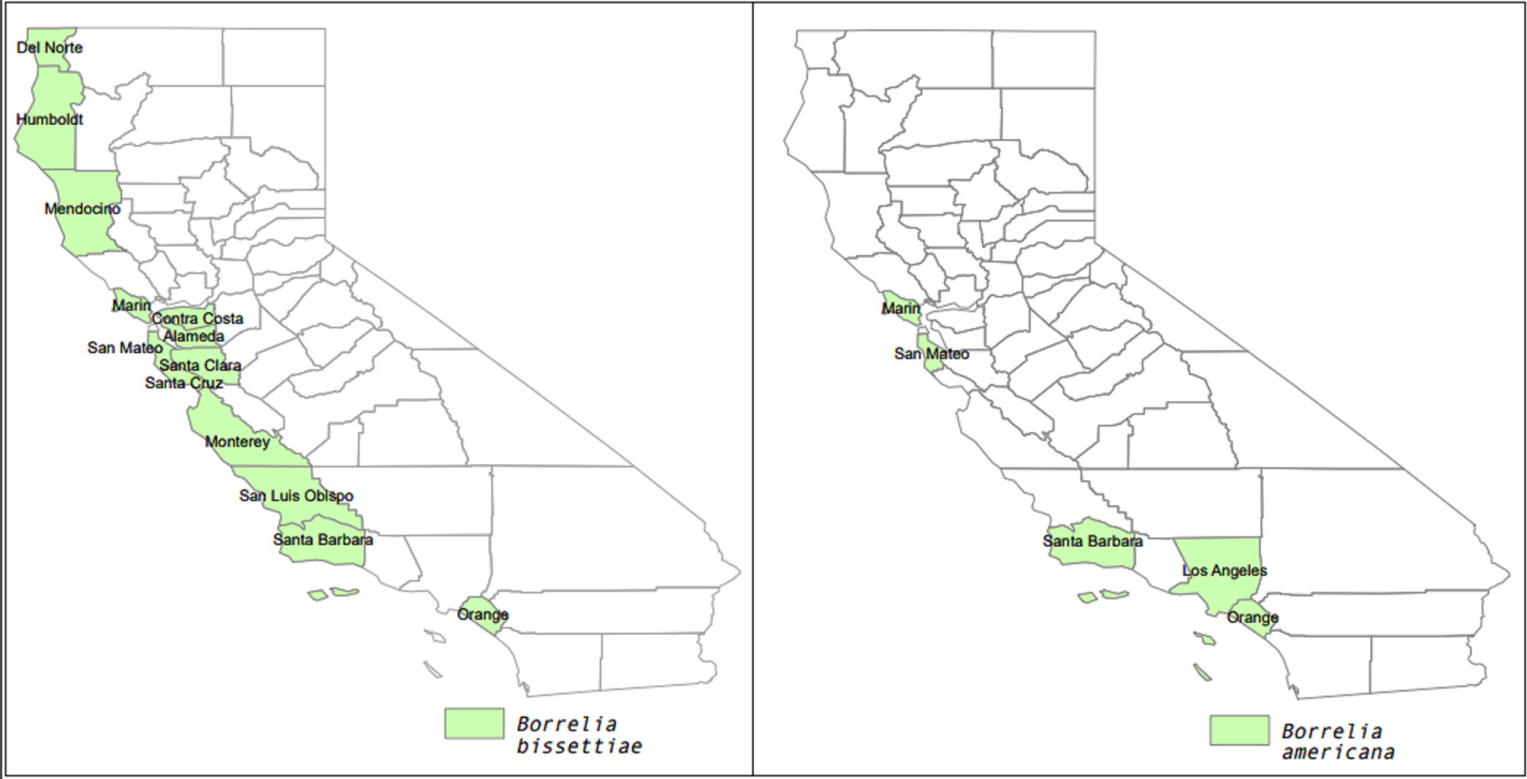
Maps showing reported presence of Borrelia bissettiae (left) and *B. americana* (right) in California counties, based on data from this study combined with previous published reports (see Table 1 for details and references). The maps were created in ArcMap, and the polygon feature class of the California county boundaries was downloaded from ArcGIS (credits: U.S. Bureau of Reclamation, California Department of Conservation, California Department of Fish and Game, California Department of Forestry and Fire Protection, and National Oceanic and Atmospheric Administration).

Multiple *Borrelia* species (*B. bissettiae*, *B. americana*, Borrelia californiensis, and B. burgdorferi *sensu stricto*) have also been observed in coastal habitats in southern California (27) (Fig. 7; Table 1). Wood rats (*Neotoma* spp.) may play a role in *Borrelia* transmission in these environments, as they have been found to be infected with *B. bissettiae*, B. miyamotoi, and B. burgdorferi (21, 22, 25). *Peromyscus* mice may also be important in these habitats (25, 27). Verification of host reservoir roles in coastal habitats requires further investigation, but the existing data suggest that *Borrelia* transmission dynamics are very different from the archetypal black oak woodland study systems, where wood rats and mice are believed to play largely peripheral roles in Lyme disease ecology (3, 5).

*B. americana* was observed in three I. pacificus ticks, all collected in coastal chaparral habitat in Marin County. Prior observations of *B. americana* in I. pacificus were also linked to chaparral/grassland habitat in San Mateo and Los Angeles counties (26, 30). In southern California, *B. americana* has also been observed in *I. spinipalpis* (26, 27). Though data are still admittedly sparse, *B. americana* has been consistently observed in grassland/chaparral habitat, presumably because its reservoir host is associated with this habitat. Human infections with *B. americana* have not been reported (39).

A single *B. bissettiae-*infected tick was recovered from Monterey County. *B. bissettiae* has been associated with wood rats (*Neotoma* spp.) and other small mammals (Table 1) and is potentially also a zoonotic pathogen, as it has been found infecting humans in northern California (20). It appears to be widely distributed in California’s coastal region (Fig. 7).

We did not observe a pattern of dominance by either B. burgdorferi or B. miyamotoi, echoing previous reports from both California and the northeastern United States (13, 40). Furthermore, based on the phylogeny, we found no evidence of geographic clustering of B. burgdorferi by latitude or sampling location (Fig. 3 and 4).

### Surveillance of adult versus nymphal ticks.

Despite our best efforts, we struggled to find nymphs in Monterey County with tick flagging. However, adult I. pacificus ticks are abundant in Monterey County, and a variety of tick-borne pathogens are present (B. burgdorferi *sensu stricto*, B. miyamotoi, *B. bissettiae*, and A. phagocytophilum). Tick flagging is regarded as a sampling method that is representative of human exposure to ticks, and if this is the case, then human exposure to nymphs is rare in Monterey County Indeed, patterns of nymphal tick submissions from citizen scientists were rare in Monterey County (and counties further south) and were seen only in May (which is when we carried out surveillance for this study) (41). In contrast, citizen scientists reported adult ticks from Monterey County for several months (and from a broader swath of California) (41).

Although nymphs are regarded as the life stage that is most responsible for Lyme disease transmission (42), adult ticks are often easier to collect in abundance due to their habit of questing on higher vegetation and because they are more noticeable on tick flags. As such, adult ticks are good sentinels to demonstrate the local presence and diversity of *Borrelia* species. We observed *B. bissettiae* and *B. americana* only in adult ticks, though this may have been due to the larger samples as well as the habitat associations that appear to be important for *B. americana* ecology; i.e., it is difficult to collect nymphs in grassland/chaparral. Given the opportunity, we recommend that both adult and nymphal stages be included in tick-borne disease surveillance in California.

## MATERIALS AND METHODS

### Field sites and tick collection.

Sampling sites were predominantly recreational areas or hiking trails, e.g., California state parks (SP) and midpeninsula open space preserves (OSP), in Marin, Mendocino, Monterey, Napa, Santa Clara, Santa Cruz, and Sonoma counties in northwest California (Table 3; Fig. 2). Some privately owned sites were also surveyed. Data are presented as belonging to a particular site which represents a single trail.

The study was conducted between December 2015 and May 2018 (Table 3; see also Data Set S1 in the supplemental material). Adult western black-legged ticks were predominantly collected each winter (December and January), when they are questing in greatest abundance (43). Collections in spring (May) were focused on nymphal ticks, though adults and larvae were also present and collected opportunistically. We attempted to visit each site during both winter and spring, but heavy rains and/or damage from wildfires precluded repeat visits in many locales. Olompali State Park was visited on three occasions.

Ticks were collected by dragging a 1-m^2^ white flannel blanket along vegetation abutting trails for 20 m; ticks that attached themselves to the flannel were removed. We also collected ticks that were observed on vegetation, as well as any ticks found crawling on clothes or skin. We recorded the GPS coordinates and habitat type for observed ticks—either at the point that the ticks were observed or when a 20-m drag was successful in finding a tick. To prevent pseudoreplication of geographic data, we discarded GPS coordinates within 1.415 km of each other (44), unless the observed tick was a different life stage recorded in a different sampling period. Habitat classifications were coarse and included (i) coastal scrub/chaparral, where dominant species are coyote brush (Baccharis pilularis), California sagebrush (Artemisia californica), coastal buckwheat (Eriogonum parvifolium), sawtooth goldenbush (Hazardia squarrosa), and poison oak (Toxicodendron diversilobum); (ii) coastal grassland/prairie, which is dominated by annual grasses and forbs, with various amounts of native perennials; (iii) redwood forest, where dominant species are coastal redwood (Sequoia sempervirens) with associated Douglas fir (Pseudotsuga menziesii) and tanoak (Lithocarpus densiflorus); and (iv) oak-bay forest, where dominant species are coast live oak (Quercus agrifolia) or other *Quercus* species, California bay (Umbellularia californica), madrone (Arbutus menziesii), California blackberry (Rubus ursinus), and poison oak. At some sites, the trailside habitat was mixed, normally combining patches of coastal chaparral and grassland that could not be separated.

Ticks were stored in 70% ethanol. All ticks were identified to species and stage levels via morphology, and here we describe only observations of ticks identified as I. pacificus. DNA was extracted from ticks following manufacturer’s protocols (DNeasy blood and tissue kit; Qiagen, Valencia, CA) and stored at −20°C until molecular analysis.

### Pathogen detection and identification.

To detect *Borrelia* pathogens, we used real-time PCR protocols described previously (17). In brief, we amplified a segment of the 16S rRNA gene of *Borrelia* sp. DNA (13), which enabled detection and classification of B. burgdorferi *sensu lato* (Lyme disease group) and B. miyamotoi (tick-borne relapsing fever group) through the detection of separate hybridization probes. Samples were considered positive if they had a cycle threshold (*C_T_*) value of <40 and logarithmic distributions on the amplification plots.

To identify *Borrelia* species and strain genotypes, we amplified and sequenced the 16S-23S intergenic spacer (IGS; *rrs-rrlA*) of a subset of the real-time PCR-positive tick samples using a nested-PCR protocol with a 25-μl reaction volume (45). The subset of *Borrelia-*positive ticks was chosen to represent as many different sites as possible across the geographical range of sampling. Prior to amplification of the inner target region, we used a 1× magnetic bead cleanup to purify and concentrate the target DNA. During this magnetic bead cleanup, targets were annealed to the beads, washed twice with 70% ethanol (EtOH) and diluted into 12.5 μl of molecular-grade H_2_O before being added to the inner PCR mixture. Amplified samples were sequenced using capillary Sanger sequencing on an ABI 3730 sequencer with both forward and reverse primers (EnGGen, Northern Arizona University). Successfully sequenced forward and reverse *Borrelia* sp. samples were trimmed, and forward/reverse reads were assembled using Geneious prime (version 2019.1.1). For phylogenic reconstruction, sequences from this study were chosen from each location and were aligned (muscle alignment using default settings) with sequences obtained from GenBank NCBI (HQ012505.1 [46], KC416410.1 [47], EU886969.1 [48], EU377803.1, and EU377801.1 [49]) using MEGAX (version 10.1.8). A phylogenic tree was constructed with MEGAX using the maximum-likelihood method and the Tamura-Nei model (50, 51).

Anaplasma phagocytophilum was detected using a previously described real-time PCR assay (52). We did not screen all ticks for A. phagocytophilum, so sample sizes differ from those for *Borrelia* sp. screening.

### Analyses.

Prevalence is reported as the percentage of ticks testing positive for the disease agent (i.e., number of positives/number tested × 100). Some analyses were restricted to sites where sample sizes were >30, as this removes the impact of considering sites with an inflated pathogen prevalence because a single positive was observed in a small sample, and this seems to be an informal threshold at which we are normally able to detect *Borrelia* if it is present (16).

Binomial proportion 95% confidence intervals were calculated using binom.test in R. We used Fisher's exact test or the chi-square test to evaluate differences among proportions (i.e., infection prevalence).

### Data availability.

Sanger sequencing data have been uploaded in the NCBI database under accession numbers MW862414 to MW862434.
